# Chronological Changes in Urine Color and Serum Iron Levels After Deferoxamine Administration in Acute Iron Poisoning

**DOI:** 10.1155/carm/9132181

**Published:** 2026-02-20

**Authors:** Hiroshi Ito, Reiichi Sugihara, Junta Imori, Shunichiro Nakao, Jotaro Tachino, Keishi Okamoto, Kazuhiro Yoneda, Hiroshi Ogura, Jun Oda

**Affiliations:** ^1^ Department of Traumatology and Acute Critical Medicine, Graduate School of Medicine, The University of Osaka, Suita, Japan, osaka-u.ac.jp

**Keywords:** case report, deferoxamine, iron poisoning, serum iron concentration, vin-rose

## Abstract

Acute iron poisoning is rare but potentially fatal. Deferoxamine (DFO) is the standard chelating agent and the appearance of “vin‐rose urine” has traditionally been considered a marker of effective chelation; however, its relationship with serum iron concentration over time remains unclear. A 17‐year‐old female patient presented with abdominal pain and acute liver dysfunction 43 h after intentional ingestion of sodium ferrous citrate (approximately 40 mg/kg of elemental iron). Continuous intravenous DFO was administered at a dose of 100 mg/kg for approximately 6 h. The urine color changed to vin‐rose within 1 h and normalized 12 h after DFO initiation. Despite the normalization of urine color, abdominal pain persisted. Serial laboratory analyses showed that serum iron levels transiently decreased, but then increased repeatedly, even after the normalization of urine color. This case provides chronological documentation of urine color changes alongside serial measurements of serum iron and ferritin, illustrating that the normalization of urine color does not necessarily indicate the normalization of serum iron levels. Urine color changes may need to be interpreted in conjunction with multiple clinical factors, clinical symptoms, and laboratory findings of acute iron poisoning.

## 1. Introduction

Iron poisoning is a rare and potentially fatal condition. In chronic transfusion‐dependent disorders such as thalassemia, iron accumulation frequently leads to iron overload, for which the iron chelator deferoxamine (DFO) is used to promote iron excretion [1]. This chelating agent is also used to treat acute iron poisoning [2]. The chelating agent DFO binds with serum iron to form water‐soluble ferrioxamine, which undergoes renal excretion and turns urine a pinkish color called “vin‐rose urine” [3]. Previous reviews have recommended that DFO should be administered for iron intoxication until the vin‐rose coloration of the urine normalizes and symptoms resolve [4]. Conversely, there are reports of patients with elevated serum iron concentrations despite normal urine coloration [5], suggesting that the normalization of urine color alone is insufficient as a criterion for discontinuing DFO therapy [6]. However, no evidence exists from clinical trials, and treatment is frequently based on clinical experience. Reports exist on images of vin‐rose‐colored urine after the administration of DFO [7]. However, to the best of our knowledge, no report has documented a chronological series of images illustrating the relationship between changes in urine color and serum iron concentrations over time.

This case report presents a chronological documentation of serum iron concentration, ferritin levels, and urinary coloration after DFO administration to assist in the identification and treatment of acute iron poisoning.

## 2. Case Presentation

A 17‐year‐old female patient was treated for depression by a psychiatrist at her local clinic. The patient had no significant family history of disease. Due to difficulties in her family environment, the patient overdosed with 40 tablets of sodium ferrous citrate. Each sodium ferrous citrate tablet contained 50 mg of elemental iron; therefore, the total ingested elemental iron dose was estimated to 2000 mg, corresponding to approximately 40 mg/kg based on the body weight of the patient (50 kg). She presented to a family psychiatrist approximately 36 h postingestion with mild spontaneous pain and tenderness in the right hypochondrium. Blood tests revealed elevated levels of aspartate aminotransferase (AST, 1008 U/L) and alanine transaminase (ALT, 1214 U/L), and a prothrombin time (PT%) of 24%, indicating impaired liver function. The patient was referred to our hospital 43 h after ingestion with suspected acute liver failure due to iron poisoning from a sodium ferrous citrate overdose. At admission, her airway was patent, respiratory rate was 23 breaths/min, maintaining an oxygen saturation of 99% (room air), blood pressure was 14.2/9.73 kPa, pulse was 94 beats/min, initial Glasgow Coma Scale score E4V5M6, she was conscious and coherent, and her body temperature was 38.5°C. Physical examination revealed mild pain and tenderness in the right hypochondrium. Initial blood test results revealed elevated AST (2649 U/L) and ALT (3023 U/L) levels and decreased PT (20%), indicating acute liver dysfunction. Arterial blood gas (room air) testing reported mildly elevated lactate levels (2.88 mmol/L), no evidence of metabolic acidosis (pH 7.42, serum bicarbonate 25.5 mmol/L), and a base excess of 1.6 mmol/L (Table 1). No gastric or intestinal drug masses/pockets or other abnormalities were reported on the abdominal computed tomography scan. No other pathologies causing acute hepatic injury were detected in the blood test results. The presenting acute hepatic injury was attributed to iron toxicity.

**TABLE 1:** Time course of laboratory parameters following admission for iron intoxication.

Laboratory parameters	Reference range (unit)	On admission	Time after initiation of deferoxamine (hours)							
			3	6	12	20	24	30	36	54
Serum										
AST	≦ 40 (U/L)	2649		2964				1445		490
ALT	≦ 40 (U/L)	3023		3417				2753		2071
GGT	8–51 (U/L)	61		49				45		48
Transferrin	2.0–3.4 (g/L)	1.97		1.91	1.82		1.84		1.79	1.9
Transferrin saturation	− (%)	76		27	55		71		73	52
Serum iron	7.3–22.7 (μmol/L)	56.3		39.2	45.2		50.7		47.4	29.7
UIBC	24.5–61.0 (μmol/L)	17.9		105.1	37.6		20.8		17.4	27.4
TIBC	40.5–74.9 (μmol/L)	74.2		144.3	82.9		71.4		64.8	57.1
Ferritin	4–123 (μg/L)	405		513	475		425		305	244
Coagulation										
PT‐%	70–125 (%)	20	19	21		33		39		55
PT‐INR		2.67	2.71	2.5		1.86		1.64		1.34
aPTT	24–39 (s)	33	30	31		30		31		32
Fibrinogen	1.5–3.5 (g/L)	2.3	1.9	1.90		2.2		2.30		2.1
D‐dimer	0.0–0.5 (μg/mL)	8.9	17.5	20.1		33.1		33.0		14.4
FDP	< 5.0 (μg/mL)	17.3	29.3	30.9		49.7		44.2		22.1
Blood gases (room air)										
pH		7.42	7.41	7.45	7.43			7.42		7.45
PaO2	11.5–14.3 (kPa)	10.7	8.9	8.9	12.6			15.5		9.5
PaCO2	4.7–6.0 (kPa)	5.3	5.4	4.8	5.3			5.4		5.1
HCO3	22–26 (mmol/L)	25.5	25.2	24.7	26.1			26.1		26
BE	− (mmol/L)	1.6	1.2	0.9	1.6			1.3		2
Lactate	0.6–2.2 (mmol/L)	2.9	2.7	2.8	1.4			1.2		1.1

*Note:* ALP: alkaline phosphatase, LDH: lactate dehydrogenase, PaO2: partial arterial pressure in oxygen, PaCO2: partial arterial pressure in carbon dioxide, HCO3: bicarbonate.

Abbreviations: ALT, alanine aminotransferase; aPTT, activated partial thromboplastin time; AST, aspartate aminotransferase; BE, base excess; FDPs, fibrin/fibrinogen degradation products; GGT, gamma‐glutamyl transferase; PT, prothrombin time; PT‐INR, prothrombin time–international normalized ratio; TIBC, total iron‐binding capacity; UIBC, unsaturated iron‐binding capacity.

**FIGURE 1:**
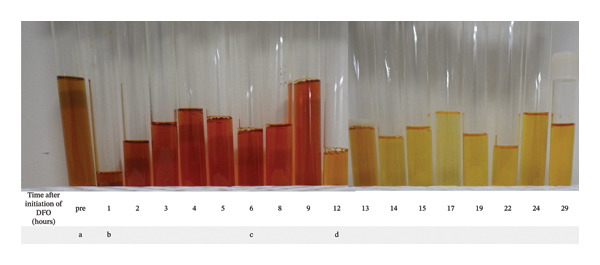
Changes in urine color before and after deferoxamine administration. DFO was administered over approximately 6 h. The urine color changed from (a) before DFO administration to (c) “vin‐rose urine” at the end of DFO administration. After commencing DFO administration, the urine color changed to normal at (d) 12 h after administration. Left to right: (a) before administration and after administration at (b) 1 h, (c) 6 h, and (d) 12 h. DFO, deferoxamine.

DFO (16 mg/kg/h) was administered as a continuous intravenous infusion to treat acute hepatic injury secondary to iron toxicity; fresh frozen plasma (FFP) was transfused to replenish coagulation factors. Before DFO administration, the urine was rusty and iron‐colored (Figure 1(a)), which changed to a vin‐rose coloration within 1 h after commencing DFO administration (Figure 1(b)). Although the recommended maximum daily dose is 80 mg/kg, a higher dose of 100 mg/kg was administered as a continuous intravenous infusion over approximately 6 hours—reaching a cumulative dose of 5000 mg—without worsening consciousness or metabolic acidosis. However, the pain in the right hypochondrium persisted, accompanied by nausea and vomiting. The color of the urine remained unchanged (Figure 1(c)). Twelve hours after the initiation of DFO administration, the urine approached a normal light‐yellow coloration (Figure 1(d)). Although right hypochondrial pain persisted, no decrease in consciousness or worsening of metabolic acidosis was observed after DFO administration. DFO was administered only during the first 6 h of hospitalization and was not continued thereafter. Fourteen units of FFP (1680 mL) were administered within 24 h of admission.

On the third day, the patient continued to experience right hypochondrial pain; however, blood test results revealed an improvement in acute hepatic injury (AST 1445 U/L and ALT 2753 U/L). The PT% was 39%, which was deemed low despite improvement; therefore, four additional units of FFP (480 mL) were administered to the patient. The hepatic injury gradually improved by Day 5, and the right hypochondrial pain disappeared. The condition of the patient stabilized and she was later transferred to another hospital where her previous physician resided 18 days after symptom onset.

**FIGURE 2:**
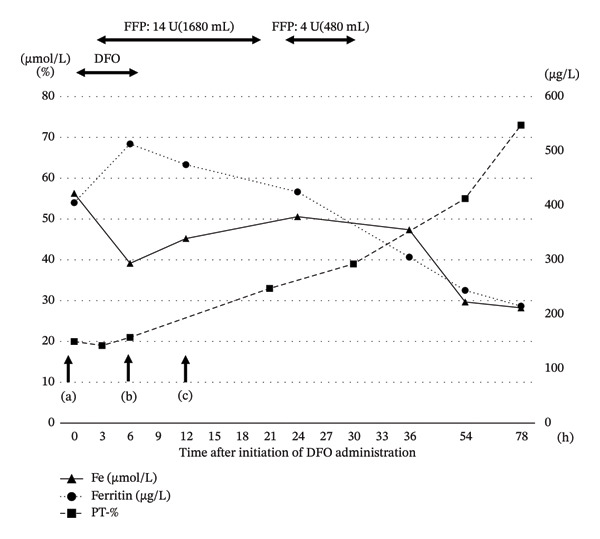
Blood test results after admission. Serum iron concentration, ferritin levels, and PT (%) after hospitalization. (a) Before DFO administration, (b) at the end of DFO administration, and (c) 12 h after administration, the urine color normalized. Serum iron concentration did not normalize within 12–54 h following DFO administration, even when urine color was normal. PT% gradually improved. DFO, deferoxamine; PT, prothrombin time; FFP, fresh frozen plasma.

The patient kept in‐hospital follow‐up on the weekends. Although PT% can be measured by emergency testing, serum iron and ferritin levels are not immediately available in acute settings. On weekdays, blood samples collected at admission were analyzed post hoc. At the commencement of treatment, serum iron levels were 56.3 μmol/L and ferritin levels were 405 μg/L (Figure 2(a)), suggesting that serum iron may have already been taken up by cells and accumulated as ferritin. Six hours after DFO administration, serum iron decreased (39.2 μmol/L) and ferritin levels increased (513 μg/L) (Figure 2(b)). After urine color changed to normal (12 h after initiation of DFO administration), serum iron concentration was 45.2 μmol/L and ferritin levels dropped to 475 μg/L (Figure 2(c)). At 24, 36, and 54 h after the initiation of DFO administration, serum iron concentrations were 50.7, 47.4, and 29.7 μmol/L, respectively, while ferritin levels were 425, 305, and 244 μg/L (Table 1).

## 3. Discussion

Iron is intracellularly toxic and disrupts the mitochondrial electron transfer system, resulting in anaerobic metabolism [8]. The liver is particularly vulnerable to iron‐induced damage due to portal circulation; hepatocytes are more prone to free radical generation owing to their high metabolic activity [9]. Iron is rapidly absorbed after oral administration and distributed from the circulating blood to the tissues. Serum iron levels should be measured within 2–6 h following oral administration [8]. Levels over 89.5 μmol/L are considered the threshold for iron toxicity in humans, but these may decline as iron redistributes intracellularly, complicating late presentations. Thus, serum iron concentration levels are unrelated to symptomatology or the severity of intoxication [2]. When evaluating a patient’s condition, multiple factors should be considered, such as dose, symptoms, time since oral administration, and radiographic findings [10]. Our patient presented 43 h postintake; thus, serum iron levels likely underestimated the severity of toxicity. Although the patient’s vital signs were stable, she experienced moderate‐to‐severe gastrointestinal symptoms (i.e., vomiting and abdominal pain), along with blood tests demonstrating acute liver dysfunction, suggestive of acute iron poisoning.

Chelation with DFO is indicated for iron toxicity treatment when serum iron concentration exceeds 89.5 μmol/L along with signs of iron toxicity (e.g., impaired consciousness, shock, vomiting, diarrhea, and metabolic acidosis) [11]. Given our patient’s symptoms and laboratory findings, treatment with DFO was initiated.

Plasma exchange or hemodialysis may be indicated if renal excretion is compromised (e.g., oliguria) or if clinical deterioration—such as progressively impaired consciousness and worsening metabolic acidosis—persists despite DFO therapy [12]. One reported case described rapid progression to impaired consciousness from acute hepatic injury within 20 h postingestion, ultimately requiring urgent liver transplantation by Day 4 [7]. As the patient was not oliguric and proper renal excretion of the chelate was expected, dialysis was not considered as the initial treatment. As the liver dysfunction and consciousness of our patient remained stable, liver transplantation was not required.

Although normalization of urine color after DFO administration has been considered an unreliable criterion for terminating therapy [6], few studies have examined in detail the relationship between posttreatment changes in urine coloration and serum iron concentration [13]. In this case, although the recommended maximum daily dose of DFO is 80 mg/kg, a dose of 100 mg/kg was administered as a continuous intravenous infusion over 6 h on the first hospital day, with a cumulative dose of 5000 mg; however, urine color had not yet normalized, and gastrointestinal symptoms persisted at the end of treatment. Thus, the additional administration of DFO should have been considered. Although the serum iron concentration decreased to 39.2 μmol/L, ferritin levels increased to 513 μg/L (Figure 2(b)), suggesting the persistence of iron requiring further chelation.

Another notable point in this case is that, although urine color normalized 12 h after DFO administration, abdominal pain persisted along with elevated iron levels (45.2 μmol/L). This was likely because the serum iron was metabolized and accumulated as ferritin at the start of administration. Even after DFO‐mediated chelation and renal excretion of iron, residual ferritin stores may continue to release iron into the circulation, resulting in persistently elevated serum iron levels due to this rebound effect. As all the administered DFO was excreted as a chelate in the urine, complete renal excretion of all circulating iron was not possible, which may have contributed to the outcome. Previous studies have demonstrated that nontransferrin‐bound iron becomes undetectable during intravenous DFO infusion but reappears within 1 h after cessation of therapy, suggesting the mobilization of pooled iron following DFO clearance [14]. Additional DFO administration might have accelerated the resolution of abdominal pain and potentially reduced the length of hospital stay.

In iron overload disorders such as thalassemia, the administration of DFO results in chelation of serum iron and subsequent urinary iron excretion [1]. Although iron is similarly excreted in the urine in cases of acute iron poisoning, reported urine color changes vary depending on the timing of treatment initiation. In a previous report, a patient who presented 1 h after ingesting ferrous nitrate had an initial serum iron concentration of 2260 μg/100 mL (405 μmol/L); chelation therapy was initiated immediately, with urine described as orange‐red during the first 24 h and amber on the second day. In that case, chelation therapy was continued for 3 days, with particularly intensive therapy—including oral, intramuscular, and continuous intravenous administration—on the first day [13]. These observations suggest that changes in urine color are influenced by multiple factors, including the ingested iron dose, time from ingestion to treatment, chelator dosage, and duration of therapy. Furthermore, the assessment of urine color is inherently subjective and dependent on the observer. Therefore, the assumption that sufficient iron has been eliminated and that DFO therapy can be safely discontinued solely based on the normalization of urine color is highly limited. Even if the urine color is normal, serum iron levels may remain elevated; therefore, it is necessary to comprehensively interpret various factors.

In conclusion, the relationship between urine coloration and serum iron levels following DFO administration in patients with iron poisoning was chronologically evaluated and pictographically illustrated. Even after urine color normalizes, iron levels may remain elevated, necessitating a comprehensive evaluation that considers both persistent symptoms and ferritin levels. Although changes in urine color are important in the treatment of iron poisoning, it is essential to carefully evaluate the condition, considering the patient’s overall state and clinical course.

## Author Contributions

Hiroshi Ito, Reiichi Sugihara, Junta Imori, Shunichiro Nakao, Jotaro Tachino, Keishi Okamoto, and Kazuhiro Yoneda provided direct patient care. Hiroshi Ito and Reiichi Sugihara reviewed the medical records and drafted the manuscript. Reiichi Sugihara and Junta Imori measured serum and collected urine samples. Hiroshi Ogura and Jun Oda contributed to the discussion of this case report.

## Funding

This work was supported by the Grant‐in‐Aid for Scientific Research from the Japan Society for the Promotion of Science (grant numbers 21K09017 and 24K12153).

## Disclosure

All authors have read the draft and approved the final manuscript.

## Ethics Statement

Both the patient and her father consented to participate in the study.

## Consent

As the patient was a minor (age < 18 years), both the patient and her father provided written informed consent for publication.

## Conflicts of Interest

The authors declare no conflicts of interest.

## Data Availability

The data and materials are available from the corresponding author upon reasonable request.
